# The Predictive Ability of MAGGIC Score After Coronary Artery Bypass Grafting: A Comparative Study

**DOI:** 10.21470/1678-9741-2022-0355

**Published:** 2023-06-14

**Authors:** Sevgi Ozcan, Esra Dönmez, Murat Ziyrek, Bülent Mert, Irfan Şahin, Ertuğrul Okuyan, Berk Özkaynak

**Affiliations:** 1 Department of Cardiology, Bağcılar Training and Research Hospital, Bağcılar, İstanbul, Turkey; 2 Department of Cardiovascular Surgery, Bağcılar Training and Research Hospital, Bağcılar, İstanbul, Turkey

**Keywords:** Coronary Artery Bypass, Area Under Curve, Heart Failure, Prognosis, Risk Assessment, Reproducibility of Results

## Abstract

**Introduction:**

The European System for Cardiac Operative Risk Evaluation (EuroSCORE) II and the Society of Thoracic Surgeons (STS) are validated scoring systems for short-term risk estimation after coronary artery bypass grafting (CABG). The Meta-Analysis Global Group in Chronic Heart Failure (MAGGIC) risk score is originally aimed to estimate mortality in heart failure patients; however, it has showed a similar power to predict mortality after heart valve surgery. In this study, we sought to evaluate whether MAGGIC score may predict short and long-term mortality after CABG and to compare its power with EuroSCORE II and STS scoring systems.

**Methods:**

Patients who underwent CABG due to chronic coronary syndrome at our institution were included in this retrospective study. Follow-up data were used to define the predictive ability of MAGGIC and to compare it with STS and EuroSCORE-II for early, one-year, and up to 10-year mortality.

**Results:**

MAGGIC, STS, and EuroSCORE-II scores had good prognostic power, moreover MAGGIC was better for predicting 30-day (area under the curve [AUC]: 0.903; 95% confidence interval [CI]: 0.871-0.935), one-year (AUC: 0.931; 95% CI: 0.907-0.955), and 10-year (AUC: 0.923; 95% CI: 0.893-0.954) mortality. MAGGIC was found to be an independent predictor to sustain statistically significant association with mortality in follow-up.

**Conclusion:**

MAGGIC scoring system had a good predictive accuracy for early and long-term mortality in patients undergoing CABG when compared to EuroSCORE-II and STS scores. It requires limited variables for calculation and still yields better prognostic power in determining 30-day, one-year, and up to 10-year mortality.

**Figure:**
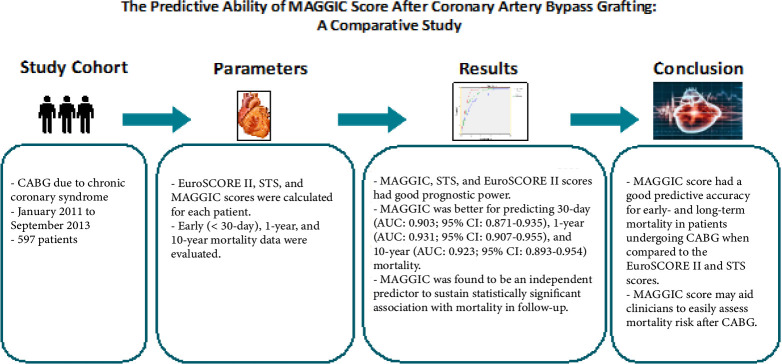

**Table:** 

Abbreviations, Acronyms & Symbols				
ACEI	= Angiotensin converting enzyme inhibitor		HT	= Hypertension
ARB	= Angiotensin receptor blocker		IQR	= Interquartile range
AUC	= Area under the curve		MAGGIC	= Meta-Analysis Global Group in Chronic Heart Failure
BMI	= Body mass index		MI	= Myocardial infarction
CABG	= Coronary artery bypass grafting		NYHA	= New York Heart Association
CAD	= Coronary artery disease		OR	= Odds ratio
CI	= Confidence interval		PAD	= Peripheral artery disease
COPD	= Chronic obstructive pulmonary disease		PCI	= Percutaneous coronary intervention
DM	= Diabetes mellitus		ROC	= Receiver operating characteristic
EF	= Ejection fraction		STS	= Society of Thoracic Surgeons
EuroSCORE	= European System for Cardiac Operative Risk Evaluation		TAVI	= Transcatheter aortic valve implantation

## INTRODUCTION

Coronary artery disease (CAD) is the leading cause of death worldwide. The management of CAD has transformed significantly as a result of improvements in both medical and surgical therapies as well as percutaneous revascularization (percutaneous coronary intervention [PCI]) techniques. Currently, PCI and coronary artery bypass grafting (CABG) are the main treatment options for revascularization in which decision is made according to the risk stratification specified by the guidelines^[1]^. With regards to CABG, it is crucial to identify risk groups to optimize perioperative care of patients undergoing cardiac surgery and their postoperative follow-up. For the short-term mortality and morbidity risk estimation, several scoring systems were developed including the most widely used European System for Cardiac Operative Risk Evaluation (EuroSCORE) II and the Society of Thoracic Surgeons (STS) scoring systems^[2-4]^. On the other hand, the Meta-Analysis Global Group in Chronic Heart Failure (MAGGIC) is a recently developed risk scoring system which originally aimed to estimate mortality in heart failure patients^[5]^. The MAGGIC score was also investigated in transcatheter aortic valve implantation (TAVI) and heart valve surgery patients which revealed to have similar power to predict mortality in heart valve surgery and was shown as an independent predictor of all-cause death in TAVI patients^[6-8]^. The EuroSCORE II involves 18 clinical and laboratory parameters while the calculation of the STS score requires as many as 65 variables, which may not always be available in daily practice. Consequently, the complexity of these conventional scores justifies the need for a pragmatic and simple risk scoring system. The MAGGIC risk score consists of 13 simple variables including age, sex, ejection fraction (EF), systolic blood pressure, body mass index (BMI), serum creatinine level, New York Heart Association (NYHA) class, smoking status, presence of heart failure, chronic obstructive pulmonary disease (COPD), and diabetes, as well as use of beta-blockers and angiotensin converting enzyme inhibitor/angiotensin receptor blocker (ACEI/ARB). The prognostic value of MAGGIC score has not been studied in CABG patients. In this study, we sought to evaluate whether MAGGIC score may predict short and long-term (30-day, one-year, and 10-year) mortality after CABG and to compare it with the validated EuroSCORE II and STS scoring systems.

## METHODS

Patients ≥ 18 years of age who have undergone CABG due to chronic coronary syndrome^[1]^ between January 2011 and September 2013, with follow-up through March 2022, at our institution were included in this retrospective study. Pre, peri, and postoperative data were retrieved from hospital database and patients’ files. Demographic, clinical, and laboratory parameters were noted for each patient. Laboratory parameters on admission were included. Patients who required emergency CABG, concurrent heart valve and/or carotid artery surgery were excluded, in addition to patients with incomplete information about postoperative hospital complications and those with lack of information crucial to calculate any of the abovementioned scores.

The Institutional Ethical Committee approved the study (2021/78. 26/10/2021), which was carried out in accordance with the ethical standards of the institutional and/or national research committee and with the Declaration of Helsinki (1964) and its later amendments or comparable ethical standards; patient consent was waived accordingly.

The EuroSCORE II (http://www.euroscore.org/calc.html), STS (https://www.sts.org/resources/risk-calculator), and MAGGIC (https://www.mdcalc.com/maggic-risk-calculator-heart-failure) risk scores were calculated for each patient using available data.

Early mortality was defined as death within 30 days after surgery. Also, one-year and up to 10-year mortality data were retrieved from the national electronic system database. Subsequently, the patients who passed away within the 1^st^ year were included in the one-year mortality group whereas those who died within 10 years after surgery were assessed as the 10-year mortality group. With regards to comparative analysis, Control I group specifies patients that survived in the first 30 days after surgery, Control II group denotes patients who survived one year after the surgery, where Control III group indicates those who survived up to 10 years after CABG.

The EuroSCORE II definitions were used for preoperative characteristics, including COPD, peripheral artery disease (PAD), critical preoperative state, left ventricular EF, and pulmonary hypertension (HT), and categories for renal impairment using creatinine clearance or dialysis^[3]^. Furthermore, the MAGGIC risk score consists of 13 simple variables including age, sex, EF, systolic blood pressure, BMI, serum creatinine level, NYHA class, smoking status, presence of heart failure, COPD, and diabetes, as well as use of beta-blockers and ACEI/ARB. Variables were retrieved from admission information. Transthoracic echocardiography was performed in all patients (Vivid S70; GE Medical System, Horten, Norway), and left ventricular EF was measured using Simpson’s method. Heart failure was graded using the NYHA functional classification^[9]^. HT was defined as prescribed medications for lowering blood pressure, any measurement > 140/90 mmHg prior to operation, and/or a previous formal diagnosis^[10]^. Stroke was defined as any history of neurological deficits lasting > 24 hours that resulted from impaired cerebral blood flow^[11]^. A fasting blood sugar level ≥ 126 mg/dL (7.0 mmol/L) or use of antidiabetic medicine was indicative of diabetes mellitus (DM)^[12]^.

The primary endpoint of this study was assessment of 30-day mortality and the secondary endpoints were one-year and up to 10-year mortality during the follow-up.

### Statistical Analysis

Continuous variables were presented as mean ± standard deviation or median and interquartile range (IQR), as appropriate. Dichotomous variables were defined as percentages and numbers. In order to stratify groups, patients were divided into two groups according to the median value of MAGGIC risk score as low and high MAGGIC groups. Chi-square test was used to compare the differences between two groups for categorical variables, and Student’s *t* test for continuous variables. The Kaplan-Meier test was used to evaluate the incidence of all-cause death after CABG, and log-rank test was used to compare the difference of survival between two MAGGIC groups. Confounders in multivariate analysis were determined based on clinical significance. Receiver operating characteristic [ROC] curve analysis was performed to examine the discriminating powers of MAGGIC, STS, and EuroSCORE risk scores. The association between the level of risk of death predicted by a score and the patient’s mortality, adjusted for the other scores, was tested by logistic regression. The calibration of the models was evaluated by the Hosmer-Lemeshow test. Statistical analysis was performed using the IBM Corp. Released 2012, IBM SPSS Statistics for Windows, version 21.0, Armonk, NY: IBM Corp. software. A *P*-value was two-sided, and a *P*-value < 0.05 was considered statistically significant.

## RESULTS

**Fig. 1:**
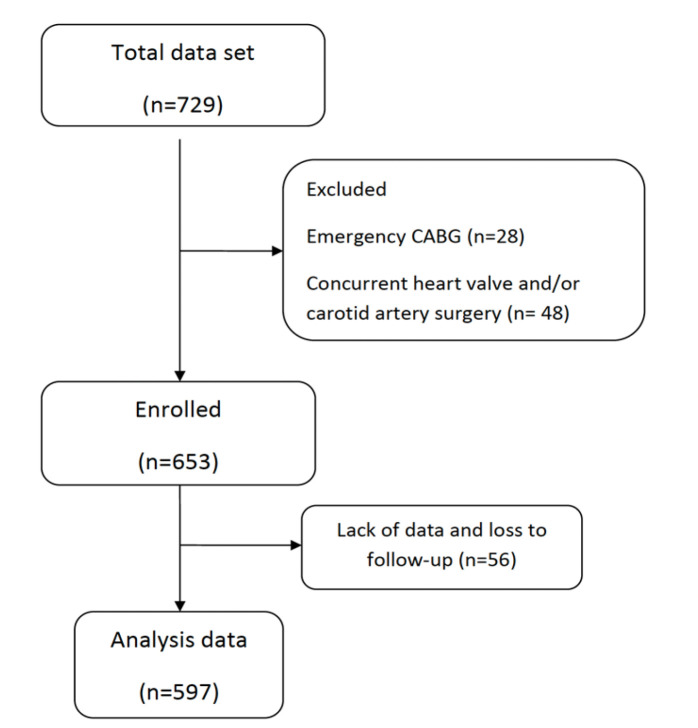
Selection of the study population. This study enrolled 729 patients. We excluded 132 patients who underwent emergency coronary artery bypass grafting (CABG), concurrent heart valve and/or carotid artery surgery, patients with missing data, and patients lost to follow-up. Finally, we analyzed 597 patients.

A total of 729 patients were evaluated and after exclusion of 132 patients who underwent emergency CABG, concurrent heart valves and/or carotid artery surgery, patients with missing data, and those lost to follow-up, finally 597 patients were analyzed (Figure 1). The mean age was 60.3±9.9 years, and 75.4% were men. Incidence of DM was 41.4%, HT was 53.3%, current smokers were 49.7%, PAD was 8.2%, carotid artery disease was 8.7%, COPD was 20.3%, and stroke was 3.7%. Patients with a history of previous myocardial infarction (MI) were 27.8%, and overall mean EF was 50.9±8.65%. There were 225 (37.6%) patients with EF < 50%. Mean EuroSCORE-II was 1.836±1.166 and STS score was 0.747±580 in overall patient population. Baseline clinical and demographic characteristics of the individuals are shown in Table 1.

**Table 1:** Patients’ characteristics.

Variables	Overall (n=59)	Low MAGGIC score (n=322)	High MAGGIC score (n=275)	*P*-value
Age (years)	60.3±9.9	55.6±8.2	65.7±8.8	< 0.001
Sex				0.301
Female, n (%)	147 (24.6%)	74 (23.0%)	73 (26.5%)	
Male, n (%)	450 (75.4%)	248 (77.0%)	202 (73.5%)	
Body mass index (kg/m^2^)	28.5±4.3	28.5±4.2	27.4±4.3	0.987
Current smoking, n (%)	297 (49.7%)	177 (54.8%)	120 (43.8%)	0.007
Family history for premature atherosclerosis, n (%)	209 (35.0%)	105 (32.5%)	104 (38.0%)	0.164
Diabetes mellitus, n (%)	247 (41.4%)	107 (33.1%)	140 (51.1%)	< 0.001
Hypertension, n (%)	318 (53.3%)	154 (47.7%)	164 (59.9%)	0.003
Peripheral artery disease, n (%)	49 (8.2%)	15 (4.7%)	34 (12.4%)	0.001
Chronic obstructive pulmonary disease, n (%)	121 (20.3%)	45 (13.9%)	76 (27.7%)	< 0.001
Stroke, n (%)	22 (3.7%)	7 (2.2%)	15 (5.5%)	0.033
Carotid artery disease, n (%)	52 (8.7%)	19 (5.9%)	33 (12.0%)	0.008
Previous myocardial infarction	166 (27.8%)	69 (21.4%)	97 (35.4%)	< 0.001
Beta-blocker use, n (%)	226 (37.9%)	143 (44.3%)	83 (30.3%)	< 0.001
ACEI/ARB use, n (%)	270 (45.2%)	157 (48.6%)	113 (41.2%)	0.072
Serum albumin (g/dL)	3.8±0.44	3.8±0.45	3.8±0.43	0.955
Serum creatinine (mg/dL)	0.97±0.64	0.86±0.21	1.1±0.89	< 0.001
Hemoglobin A1c (%)	6.4±1.97	6.3±2.0	6.6±1.9	0,084
Left ventricular ejection fraction (%)	50.9±8.65	52.2±7.98	49.4±9.17	< 0.001
NYHA class III/IV	158 (26.4%)	53 (16.4%)	105 (38.3%)	< 0.001
STS score	0.747±0.580	0.507±0.290	1.029±0.698	< 0.001
EuroSCORE II	1.836±1.166	1.399±0.741	2.351±1.352	< 0.001

ACEI/ARB=angiotensin converting enzyme inhibitor/angiotensin receptor blocker; EuroSCORE=European System for Cardiac Operative Risk Evaluation; MAGGIC=Meta-Analysis Global Group in Chronic Heart Failure; NYHA=New York Heart Association; STS=Society of Thoracic Surgeons

Median MAGGIC risk score was 16 (IQR: 4-35). Patients with high MAGGIC score had significantly higher rates of advanced age, advanced NYHA class, smoking habit, DM, HT, COPD, PAD, carotid artery disease, previous MI, stroke, lower EF, higher serum creatinine levels, and high EuroSCORE II and STS scores when compared to those with low MAGGIC score. However, beta-blocker usage was more common in the low MAGGIC score group (44.3 *vs.* 30.3%, *P*<0.001).

A total of 40 (6.7%) patients were in the early-mortality group, where the surviving patients formed the Control I group. Clinical characteristics of the two groups were shown in Table 2. The early-mortality group showed older age and increased rates of DM, carotid artery disease, advanced NYHA class, as well as increased hemoglobin A1c and serum creatinine levels, STS score (0.701±0.510 *vs.* 1.381±0.995, *P*<0.001), EuroSCORE II (1.739±1.094 *vs.* 3.187±1.306, *P*<0.001), and MAGGIC score (15.96±4.99 *vs.* 24.1±3.69, *P*<0.001); however, it showed lower beta-blocker and ACEI/ARB usage. Logistic regression analysis was performed to determine independent risk factors of early mortality. The results showed that advanced age, lower beta-blocker usage, and higher MAGGIC score were independent risk factors for early mortality. Data regarding regression analysis are presented in Table 3. In ROC analyses, a cutoff value of 20.5 for MAGGIC score was associated with 83% sensitivity and 84% specificity (area under the curve [AUC]: 0.903; 95% confidence interval [CI]: 0.871-0.935) in prediction of early mortality (Figure 2A).

**Table 2:** Patients’ characteristics according to early mortality.

Variables	Overall (n=59)	Survival	Early mortality	P-value
Age (years)	60.3±9.9	59.9±9.8	64.8±9.3	0.003
Sex				0.234
Male, n (%)	450 (75.4%)	423 (76.0%)	27 (67.5%)	
Female, n (%)	147 (24.6%)	134 (24.0%)	13 (32.5%)	
Body mass index (kg/m^2^)	28.5±4.3	28.5±4.2	27.4±4.3	0.786
Current smoking, n (%)	297 (49.7%)	282 (50.6%)	15 (37.5%)	0.109
Family history for premature atherosclerosis, n (%)	209 (35.0%)	194 (34.8%)	15 (37.5%)	0.732
Diabetes mellitus, n (%)	247 (41.4%)	222 (39.9%)	25 (62.5%)	0.005
Hypertension, n (%)	318 (53.3%)	295 (53.0%)	23 (57.5%)	0.578
Peripheral artery disease, n (%)	49 (8.2%)	44 (7.9%)	5 (12.5%)	0.310
Chronic obstructive pulmonary disease, n (%)	121 (20.3%)	111 (19.9%)	10 (25.0%)	0.441
Stroke, n (%)	22 (3.7%)	19 (3.4%)	3 (7.5%)	0.185
Carotid artery disease, n (%)	52 (8.7%)	44 (7.9%)	8 (20.0%)	0.009
Previous myocardial infarction, n (%)	166 (27.8%)	154 (27.6%)	12 (30.0%)	0.748
Beta-blocker use, n (%),	226 (37.9%)	225 (40.4%)	1 (2.5%)	< 0.001
ACEI/ARB use n (%)	270 (45.2%)	259 (46.5%)	11 (27.5%)	0.02
Serum albumin (g/dL)	3.8±0.44	3.8±0.45	3.9±0.40	0.353
Serum creatinine (mg/dL)	0.97±0.64	0.94±0.54	1.39±1.36	< 0.001
Hemoglobin A1c (%)	6.4±1.97	6.4±1.9	7.0±2.5	0.042
Left ventricular ejection fraction (%)	50.9±8.65	51.1±8.6	48.4±8.99	0.058
NYHA class III/IV, n (%)	158 (26.4%)	135 (24.2%)	23 (57.5%)	< 0.001
STS score	0.747±0.580	0.701±0.510	1.381±0.995	< 0.001
EuroSCORE II	1.836±1.166	1.739±1.094	3.187±1.306	< 0.001
MAGGIC score	15.96±4.99	15.96±4.99	24.1±3.69	< 0.001

ACEI/ARB=angiotensin converting enzyme inhibitor/angiotensin receptor blocker; EuroSCORE=European System for Cardiac Operative Risk Evaluation; MAGGIC=Meta-Analysis Global Group in Chronic Heart Failure; NYHA=New York Heart Association; STS=Society of Thoracic Surgeons

**Table 3:** Logistic regression analysis for early mortality predictors.

Variables	*P*-value	OR (95% CI)
Age	0.015	0.927 (0.873-0.985)
Hemoglobin A1c	0.957	1.006 (0.814-1.243)
Diabetes mellitus	0.985	1.009 (0.377-2.706)
Carotid artery disease	0.942	0.956 (0.280-3.267)
NYHA class III/IV	0.913	1.052 (0.421-2.630)
Beta-blocker use	0.013	0.063 (0.007-0.562)
ACEI/ARB use	0.793	0.891 (0.376-2.111)
STS score	0.592	1.178 (0.646-2.148)
EuroSCORE II	0.064	1.471 (0.978-2.214)
MAGGIC score	0.000	1.328 (1.168-1.509)
Serum creatinine	0.419	1.170 (0.799-1.713)
Left ventricular ejection fraction	0.431	1.019 (0.972-1.068)

ACEI/ARB=angiotensin converting enzyme inhibitor/angiotensin receptor blocker; CI=confidence interval; EuroSCORE=European System for Cardiac Operative Risk Evaluation; MAGGIC=Meta-Analysis Global Group in Chronic Heart Failure; NYHA=New York Heart Association; OR=odds ratio; STS=Society of Thoracic Surgeons

**Fig. 2A:**
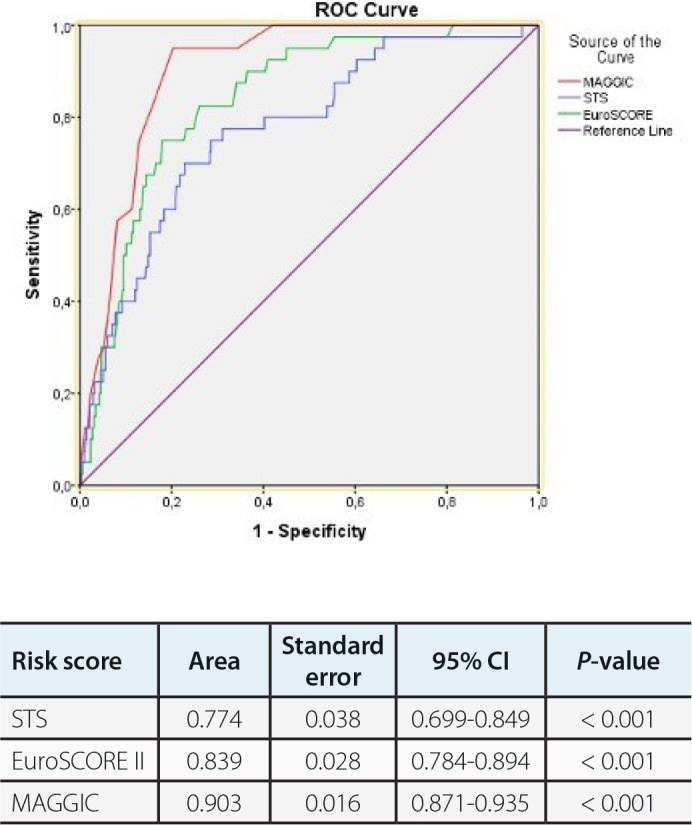
Receiver operating characteristic (ROC) curves of Meta-Analysis Global Group in Chronic Heart Failure (MAGGIC), Society of Thoracic Surgeons (STS), and European System for Cardiac Operative Risk Evaluation (EuroSCORE) II risk scores for predicting early mortality. CI=confidence interval.

There were 68 (5.03%) patients in the one-year mortality group. When the one-year mortality *vs.* Control II group (survived) were compared, the one-year mortality group showed older age, higher DM, carotid artery disease, stroke, and COPD frequencies, advanced NYHA class, lower BMI and EF, and increased serum creatinine and hemoglobin A1c levels, STS score (0.663±0.459 *vs.* 1.395±0.924, *P*<0.001), EuroSCORE II (1.664±0.985 *vs.* 3.172±1.552, *P*<0.001), and MAGGIC score (15.49±4.59 *vs.* 24.41±3.79, *P*<0.001), but lower beta-blocker and ACEI/ARB usage. In order to assess independent risk factors of death within the 1^st^ year, logistic regression analysis was performed, which showed that advanced age, lower rates of beta-blocker usage, and higher MAGGIC score, as well as higher EuroSCORE II were independent risk factors for one-year mortality. Regression analysis data are presented in Table 4. In ROC analyses, a cutoff value of 20.5 for MAGGIC score was associated with 86.8% sensitivity and 88.3% specificity (AUC: 0.931; 95% CI: 0.907-0.955) in predicting one-year mortality (Figure 2B).

**Table 4:** Logistic regression analysis for one-year mortality predictors.

Variables	*P*-value	OR (95% CI)
Age	0.002	0.917 (0.869-0.968)
Diabetes mellitus	0.662	0.821 (0.338-1.990)
Carotid artery disease	0.952	1.035 (0.338-3.166)
NYHA class III/IV	0.730	0.863 (0.374-1.991)
Beta-blocker use	0.030	0.270 (0.082-0.884)
ACEI/ARB use	0.755	0.884 (0.408-1.916)
STS score	0.687	1.132 (0.619-2.069)
EuroSCORE	0.048	1.478 (1.003-2.179)
MAGGIC score	0.000	1.556 (1.369-1.769)
Serum creatinine	0.710	0.924 (0.611-1.398)
Hemoglobin A1c	0.878	1.016 (0.830-1.244)
Left ventricular ejection fraction	0.940	1.002 (0.960-1.045)
Stroke	0.138	2.840 (0.715-1.277)
Chronic obstructive pulmonary disease	0.167	0.540 (0.225-1.293)

ACEI/ARB=angiotensin converting enzyme inhibitor/angiotensin receptor blocker; CI=confidence interval; EuroSCORE=European System for Cardiac Operative Risk Evaluation; MAGGIC=Meta-Analysis Global Group in Chronic Heart Failure; NYHA=New York Heart Association; OR=odds ratio; STS=Society of Thoracic Surgeons

**Fig. 2B:**
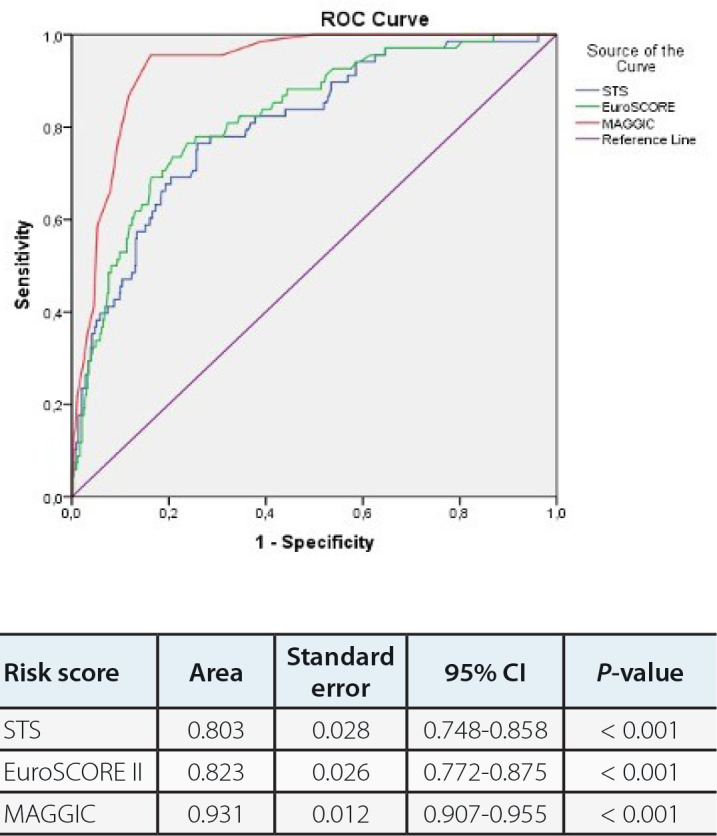
Receiver operating characteristic (ROC) curves of Meta-Analysis Global Group in Chronic Heart Failure (MAGGIC), Society of Thoracic Surgeons (STS), and European System for Cardiac Operative Risk Evaluation (EuroSCORE) II risk scores for predicting one-year mortality. CI=confidence interval.

There were 97 (17.4%) patients who were lost within the first decade after surgery. When the up to 10-year mortality group was compared to the Control III (survived) group, the former showed older age and increased rates of DM, carotid artery disease, PAD, COPD, previous MI, HT, presence of critical left main coronary artery stenosis, advanced NYHA class, lower BMI and EF, and increased serum creatinine level, smoking habit, STS score (0.599±0.390 *vs.* 1.187±0.702, *P*<0.001), EuroSCORE II (1.561±0.918 *vs.* 2.582±1.428, *P*<0.001), and MAGGIC score (14.58±3.96 *vs.* 22.55±4.1, *P*<0.001), but less beta-blocker usage. Logistic regression analysis was carried out to determine independent risk factors of 10-year mortality and showed only lower beta-blocker usage (*P*=0.028) and higher MAGGIC score were independent risk factors for 10-year mortality. Detailed results are presented in Table 5. In ROC analyses, a cutoff value of 18.5 for MAGGIC score was associated with 84.5% sensitivity and 87.6% specificity (AUC: 0.923; 95% CI: 0.893-0.954) in prediction of 10-year mortality (Figure 2C).

**Table 5:** Logistic regression analysis for follow-up mortality predictors.

Variables	*P*-value	OR (95% CI)
Age	0.568	1.017 (0.960-1.077)
Beta-blocker use	0.028	0.422 (0.196-0.910)
STS score	0.712	1.174 (0.500-2.755)
EuroSCORE	0.766	0.943 (0.640-1.389)
MAGGIC score	0.000	1.492 (1.311-1.697)
Severe left main coronary artery stenosis	0.561	1.307 (0.529-3.226)
Serum creatinine	0.077	1.486 (0.958-2.306)
Chronic obstructive pulmonary disease	0.399	1.379 (0.654-2.911)
Peripheral artery disease	0.204	1.943 (0.697-5.413)
Diabetes mellitus	0.479	0.761 (0.358-1.620)
Carotid artery disease	0.987	0.991 (0.326-3.015)
Current smoking	0.620	0.833 (0.405-1.714)
Left ventricular ejection fraction	0.334	0.981 (0.942-1.020)
Body mass index	0.230	0.950 (0.874-1.033)
NYHA class III/IV	0.627	1.232 (0.532-2.853)
Previous myocardial infarction	0.705	1.153 (0.551-2.416)
Hypertension	0.077	1.486 (0.958-2.306)

CI=confidence interval; EuroSCORE=European System for Cardiac Operative Risk Evaluation; MAGGIC=Meta-Analysis Global Group in Chronic Heart Failure; NYHA=New York Heart Association; OR=odds ratio; STS=Society of Thoracic Surgeons

**Fig. 2C:**
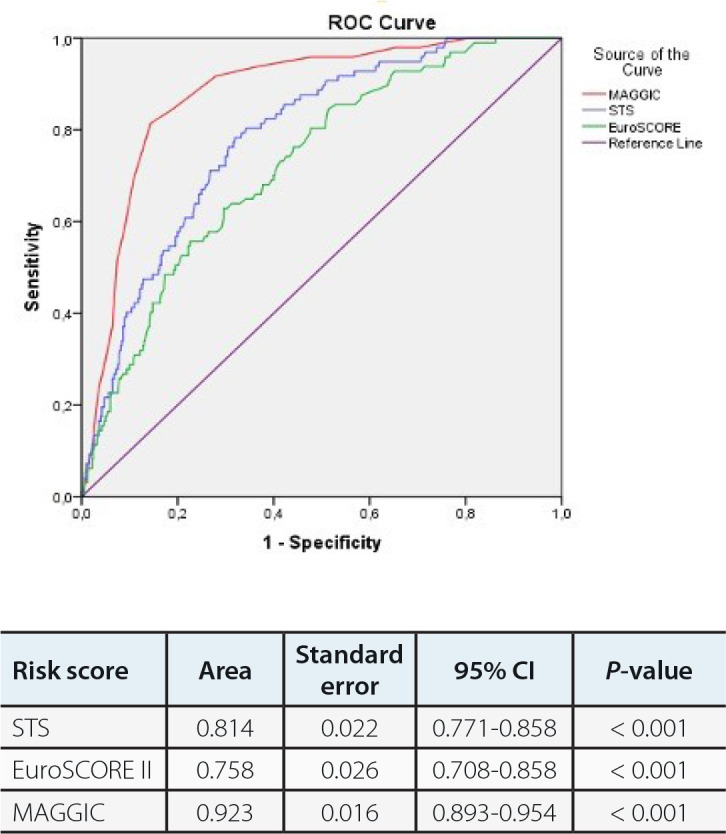
Receiver operating characteristic (ROC) curves of Meta-Analysis Global Group in Chronic Heart Failure (MAGGIC), Society of Thoracic Surgeons (STS), and European System for Cardiac Operative Risk Evaluation (EuroSCORE) II risk scores for predicting mortality up to 10 years. CI=confidence interval.

The Kaplan-Meier test demonstrated that the high MAGGIC risk score was associated with higher mortality as compared to low MAGGIC risk score either for early and follow-up mortality (14.6% vs. 0%, log-rank *P*<0.001; 38.9% *vs.* 1.9%, log-rank *P*<0.001, respectively) (Figures 3A and 3B).

**Fig. 3A:**
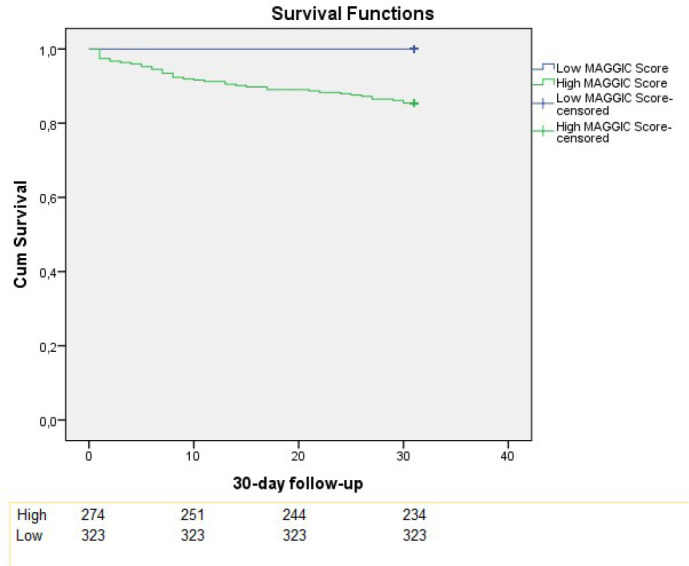
Kaplan-Meier survival curves for early mortality. MAGGIC=Meta-Analysis Global Group in Chronic Heart Failure.

**Fig. 3B:**
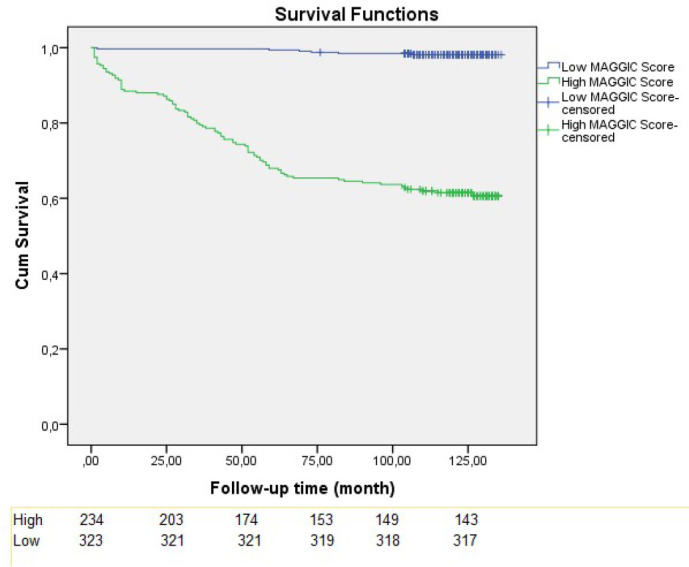
Kaplan-Meier survival curves for follow-up mortality. MAGGIC=Meta-Analysis Global Group in Chronic Heart Failure.

## DISCUSSION

To our best knowledge, this is the first study designed to assess clinical validation of the MAGGIC risk score to predict all-cause death after CABG. This single-center retrospective study showed that MAGGIC, STS, and EuroSCORE scores had good prognostic power, and that MAGGIC score was better for predicting all-cause 30-day, one-year, and 10-year mortality risk. MAGGIC was found to be independent predictor to sustain statistically significant association with mortality in follow-up according to regression analyses.

STS score and EuroSCORE II are validated and widely used risk scores to predict perioperative morbidity and mortality after cardiac surgery. Whereas, both scoring systems consist of multiple variables that may not be readily available, such as coronary artery anatomy or valve pathologies on echocardiography for STS score and presence and specific degree of pulmonary HT or extracardiac arteriopathy for EuroSCORE II^[3,4]^. For these reasons, these missing variables negatively affect the predictive ability of STS score and EuroSCORE II for perioperative risk estimation^[13]^. Stratifying high risk patients who require close monitoring are crucial for patient management and to raise the assignments of sources.

In this context, MAGGIC can be a viable alternative to these established risk prediction models in CABG. MAGGIC, with only 13 key demographic variables, provides a comparatively simple and user-friendly tool for clinicians, qualities that can extend its usefulness beyond its limits, the original heart failure population from which it is derived^[8,14,15]^. However, its prognostic importance has also been demonstrated in various cardiac diseases other than heart failure. The MAGGIC risk score has been identified as a valid prognostic model for patients after aortic and mitral valve surgery, with fewer variables considering its potential advantages over STS score and EuroSCORE II^[7]^. In another study, the MAGGIC score predicted all-cause death, especially in the transcatheter aortic valve replacement population with a high risk of STS^[6]^. The present study contributed more on the substantial literature by demonstrating the novel benefit of MAGGIC risk score in CABG patients. Patients with a high MAGGIC risk score compared to the patients in the lower values were demonstrated to have higher risk of short and long-term death. Age, EF, systolic blood pressure, BMI, creatinine level, NYHA class, sex, history of DM, COPD, smoking status, diagnosis of heart failure (≥ 18 months), and use of beta-blockers and ACEI/ARB are variables obligatory to calculate MAGGIC score. Age, EF, and renal function are known risk factors for CABG surgery^[16-20]^. Patients with DM tend to have advanced CAD, and CABG is a broadly applied treatment. However, short-term procedural success rates are similar, death and adverse cardiac events are more common in diabetic patients after CABG surgery^[21,22]^. COPD is a common condition in cardiac patients and was found to be related with increased postoperative complications and early death in severe cases^[23,24]^. Since these are the main components of MAGGIC score, this score offers to evaluate most of the main risk factors in a simple way. Additionally, MAGGIC risk score evaluates patient’s beta-blocker and ACEI/ARB usage, which are cornerstone of heart failure therapy. Beta-blockers are also recommended in treatment of CAD to reduce mortality, arrythmia, and ischemic events. Patients on beta-blocker or antihypertensive therapy, including ACEI/ARB, are in the lower risk for mortality according to our results. Moreover, lower beta-blocker usage was detected as an independent risk factor related with 10-year mortality. This finding emphasizes the importance of adequate use of guideline directed treatment. CAD severity and location are factors related with CABG success that are evaluated both in EuroSCORE II and STS scores. MAGGIC risk score was found to foresee mortality both at the early period and follow-up better than EuroSCORE II and STS scores, although it does not include the variable regarding coronary anatomy. This finding may emphasize the importance of patient-related hemodynamic factors and comorbidities. Likewise, experience of surgeon and hospital volume are inevitable factors that may alter the success of procedure, but these factors are nonapplicable to any risk score model^[25,26]^.

The EuroSCORE II and STS scores were designed for in-hospital risk prediction, however the studies for their power for long-term mortality estimation have shown that their predictive ability is still acceptable for two years, but decreases year by year after that^[27]^. These scores were based on collected data at the beginning of 1990s, nevertheless, patient characteristics and surgical techniques changed over time. Another issue is that the study population consisted of both elective and urgent/emergency cases, however, we excluded emergency cases and evaluated patients who are candidate for elective CABG. According to our results, MAGGIC score was better than either EuroSCORE II and STS scores in terms of predicting mortality at early stage and follow-up both at one year and 10 years after CABG, at the same time EuroSCORE II was found to be an independent predictor of mortality in one-year mortality but not in follow-up. Our results indicating early mortality were a higher than expected incidence^[28,29]^. We collected data between 2011 and 2013. The surgical revascularization techniques, postoperative care, hospital volume, and surgeons’ experience have changed significantly over time and this may be related with high early mortality observed in our study.

### Limitations

First, this is a single-center retrospectively designed study; multicenter and prospectively designed studies would be better to avoid selection or definition bias. Second, the term 30-day mortality includes mortality events both in hospital and after discharge at the 30th postoperative day. However, separating the events may help risk scores to predict them. And third, postoperative complications could not be evaluated. Patients presenting with acute coronary syndromes are usually high-risk patients and may present with cardiogenic shock. We excluded those patients, however, prospective studies including acute coronary syndrome patients will give additional information in this subgroup. Also, our study population mostly involved low-risk patients according to EuroSCORE II and STS scores; designing prospective studies including higher risk patients would be more informative.

## CONCLUSION

The results of this study indicated that the MAGGIC scoring system, which has been originally developed for the prediction of mortality in heart failure patients, also had a good predictive accuracy for early and long-term mortality in patients undergoing CABG when compared to the EuroSCORE II and STS scoring systems. Besides, the MAGGIC score requires limited variables for calculation and still yields better prognostic power in determining 30-day, one-year, and up to 10-year mortality. Thus, the MAGGIC score may aid clinicians to easily assess mortality risk in these patients. However, further studies with a larger patient population, particularly those with high risk, are needed to validate this scoring system.

**Table:** 

Authors’ Roles & Responsibilities	
SÖ	Substantial contributions to the conception or design of the work; or the acquisition, analysis, or interpretation of data for the work; drafting the work or revising it critically for important intellectual content; final approval of the version to be published
ED	Substantial contributions to the conception or design of the work; or the acquisition, analysis, or interpretation of data for the work; drafting the work or revising it critically for important intellectual content; final approval of the version to be published
MZ	Substantial contributions to the conception or design of the work; or the acquisition, analysis, or interpretation of data for the work; drafting the work or revising it critically for important intellectual content; final approval of the version to be published
BM	Substantial contributions to the conception or design of the work; or the acquisition, analysis, or interpretation of data for the work; final approval of the version to be published
İŞ	Substantial contributions to the conception or design of the work; or the acquisition, analysis, or interpretation of data for the work; final approval of the version to be published
EO	Drafting the work or revising it critically for important intellectual content; final approval of the version to be published
BÖ	Drafting the work or revising it critically for important intellectual content; final approval of the version to be published
